# High-Density Genetic Mapping Identifies QTL and Candidate Genes for Plant Architecture and Kernel Traits in Cultivated Peanut

**DOI:** 10.3390/genes17070792

**Published:** 2026-07-12

**Authors:** Yuzhuo Xia, Zhenzhen Zhang, Xianfeng Lin, Chaohuan Wang, Youlin Xia, Jinxiong Mao, Qing Du, Ming Luo, Yu You

**Affiliations:** 1 College of Agronomy and Biotechnology, Southwest University, Chongqing 400715, China; 2 Nanchong Academy of Agricultural Sciences, Nanchong 637000, China; 3 Nanchang Municipal Bureau of Agriculture and Rural Affairs, Nanchang 637000, China

**Keywords:** peanut, *Arachis hypogaea*, QTL mapping, SLAF-seq, genetic linkage map, plant architecture, kernel traits, candidate genes

## Abstract

Background/Objectives: Plant architecture and kernel-related traits are important determinants of yield potential and breeding value in peanut (*Arachis hypogaea* L.). This study aimed to construct a high-density genetic linkage map, identify quantitative trait loci (QTL) associated with these traits, and prioritize candidate genes underlying key genomic regions in cultivated peanut. Methods: A recombinant inbred line population derived from Luojiangjiwo, a sprawling large-pod line, and Fuhuasheng, an erect small-pod line, was used to construct a high-density genetic linkage map and identify QTL associated with plant architecture and kernel traits. Results: Specific-locus amplified fragment sequencing generated 1,295,490,603 clean reads, with an average Q30 of 93.67%. After SNP discovery, filtering, and linkage analysis, 2646 SNP markers were mapped to 20 linkage groups, spanning 1338.86 cM with an average marker interval of 0.51 cM. Phenotypic evaluation of 16 traits revealed broad variation among 200 recombinant inbred lines, with strong positive correlations among pod-size traits and among kernel-size traits. Composite interval mapping detected eight QTL distributed on chr04, chr05, chr13, and chr15, including five QTL for plant architecture traits and three QTL for kernel-related traits. qLBL13 for lateral branch length explained the highest phenotypic variation, whereas qMKL05 for mean kernel length was delimited to a 0.151 Mb interval containing only nine genes. Candidate-gene analysis prioritized *AH05G29360*, encoding a knotted-1-like homeobox protein; *AH05G29380*, encoding mitogen-activated protein kinase kinase 9; *AH05G29350*, encoding COP1-interacting protein 7; and *AH05G29410*, encoding a pentatricopeptide repeat-containing protein. Additional candidates included *AH15G16520*, *AH15G16460*, *AH15G16630*, and *AH15G16770* in the shared qHKW15/qMKW15 interval. Conclusions: This study identified genomic regions and biologically relevant candidate genes associated with plant architecture and kernel-related traits in peanut. These findings provide valuable genomic resources for future functional validation and facilitate marker-assisted breeding for improved plant architecture and kernel characteristics.

## 1. Introduction

Peanut (*Arachis hypogaea* L.) is an important oilseed and food legume crop cultivated widely for edible oil, protein, and other nutritional components [1,2]. Cultivated peanut is an allotetraploid species with a large and complex genome, and its polyploid origin historically limited the development of high-resolution genetic tools [3]. The release of chromosome-scale reference genomes for cultivated peanut has substantially improved genome-assisted research by providing a framework for marker development, linkage-map construction, QTL mapping, comparative genomics, and candidate-gene discovery. These genome resources have clarified the structure and evolution of the A and B subgenomes of cultivated peanut and now support more precise dissection of agronomically important traits [4,5,6].

Increasing yield remains a central objective in peanut breeding [7,8]. Yield formation in peanut is complex and depends on multiple component traits, including pod number, pod size, kernel size, seed weight, shelling percentage, and plant architecture [9]. Pod- and kernel-related traits such as pod length, pod width, hundred-pod weight, hundred-kernel weight, kernel length, and kernel width directly influence productivity and commercial quality [10]. At the same time, plant-type traits, including main stem height, lateral branch length, branch number, branch angle, and spreading or erect growth habit, affect canopy structure, planting density, light interception, field management, and suitability for mechanized production [11]. Because these traits are generally quantitative and influenced by both genetic and environmental factors, their genetic improvement through conventional phenotypic selection alone can be inefficient [12].

QTL mapping has become an important approach for dissecting the genetic architecture of complex peanut traits. Recombinant inbred line populations are particularly useful for QTL analysis because they are genetically stable, can be evaluated repeatedly across environments, and allow integration of phenotypic data with dense molecular maps. In peanut, previous studies have identified QTL associated with pod and seed traits using RIL populations and high-density marker systems. For example, Chavarro et al. used a Tifrunner × NC3033 RIL population to identify QTL for pod and seed traits relevant to peanut market classes, including pod area, pod weight, and seed weight [13]. More recently, Fang et al. reported co-localized QTL for pod and kernel traits on chromosome A05 and developed a molecular marker associated with kernel weight, demonstrating the potential of QTL mapping for marker-assisted selection in peanut breeding [10].

Despite recent progress, the genetic basis of peanut plant architecture remains less fully resolved compared with pod and kernel traits. Plant height and branching-related traits are important for ideotype breeding because they influence biomass distribution, reproductive development, and production efficiency. Zhang et al. identified QTL for plant height and branching-related traits in cultivated peanut and reported major and stable loci with potential value for improving peanut architecture [14]. Branch angle is another important architectural trait because it contributes to the distinction between spreading and erect growth types. Fine-mapping work has identified a QTL and candidate genes associated with lateral branch angle on chromosome B05, indicating that plant-type traits can be resolved into defined genomic regions for further functional validation [15].

Despite these advances, many QTL for peanut pod, kernel, and plant-type traits remain population-specific, environment-dependent, or mapped to relatively broad genomic intervals. Additional studies using mapping populations derived from parents with contrasting phenotypes are therefore needed to identify new loci, validate previously reported genomic regions, and provide candidate genes for molecular breeding. Reduced-representation sequencing approaches, including SLAF-seq and related genotyping-by-sequencing strategies, allow efficient discovery of large numbers of polymorphic markers and construction of high-density genetic maps, which can improve QTL resolution in species with complex genomes.

In the present study, a recombinant inbred line population derived from Luojiangjiwo, a sprawling large-pod peanut line, and Fuhuasheng, an erect small-pod peanut line, was used to investigate the genetic basis of pod-, kernel-, and plant-type-related traits. Therefore, this study aimed to construct a high-density SNP-based linkage map for the Luojiangjiwo × Fuhuasheng RIL population, identify QTL associated with plant architecture and kernel-related traits, and prioritize candidate genes within QTL intervals for future validation.

## 2. Materials and Methods

### 2.1. Plant Materials and Mapping Population

A recombinant inbred line population of cultivated peanut (*A. hypogaea* L.) was used to identify quantitative trait loci associated with pod, kernel, and plant architecture-related traits. The population consisted of 200 recombinant inbred lines derived from a cross between Luojiangjiwo and Fuhuasheng. Luojiangjiwo is a sprawling-type peanut line with relatively large pods, whereas Fuhuasheng is an erect-type peanut line with relatively small pods. Mature leaf samples were collected from the parental lines and the RIL population for reduced-representation genome sequencing. The study was designed to construct a high-density genetic linkage map and detect QTL associated with yield-related and plant-type traits in cultivated peanut.

### 2.2. Phenotypic Evaluation

The RIL population and two parental lines were evaluated for plant architecture, pod, and kernel traits. Sixteen phenotypic traits were recorded, including main stem height, lateral branch length, branch number, primary branch number, expansion radius, branch angle, 100-pod weight, pod length/width ratio, mean pod length, mean pod width, shell thickness, 100-kernel weight, kernel length/width ratio, mean kernel length, mean kernel width, and kernel thickness. The RIL population and the two parental lines were evaluated under field conditions in a randomized complete block design with three biological replicates. Each RIL was grown in one plot per replicate, and phenotypic measurements were recorded from ten representative plants in each plot. The mean values of the ten plants were calculated for each replicate, and the average across the three replicates was used for subsequent statistical analyses and QTL mapping.

Plant architecture traits were measured at the mature growth stage. The main stem’s height was measured as its vertical height. Lateral branch length was measured from the base of the lateral branch to the terminal growing point. The branch number and primary branch number were recorded by counting the visible branches on each plant. The expansion radius was measured as the horizontal spread of the plant canopy. Branch angle was measured as the angle between the main stem and the lateral branch.

Pod and kernel traits were measured after harvest using mature pods and kernels. The 100-pod weight and 100-kernel weight were determined from representative mature samples. Pod length, pod width, shell thickness, kernel length, kernel width, and kernel thickness were measured using representative mature pods or kernels. Length/width ratios were calculated from the corresponding length and width measurements. Phenotypic values were used for descriptive statistical analysis and QTL mapping.

### 2.3. DNA Extraction and SLAF Library Construction

Genomic DNA was extracted from peanut leaf tissue of the two parental lines and all RIL individuals. DNA quality and concentration were evaluated before library construction. Specific-locus amplified fragment sequencing was used to generate genome-wide polymorphic markers. The peanut reference genome was used for in silico enzyme digestion, and the restriction enzyme combination RsaI + HaeIII was selected for library construction. Specific-locus amplified fragment sequencing (SLAF-seq) was used to generate genome-wide polymorphic markers following the general strategy described by Sun et al. [16]. Fragments of 314–344 bp were selected as SLAF tags.

For each sample, genomic DNA was digested with the selected restriction enzymes. The digested fragments were subjected to 3′ A-tailing, ligated with dual-index sequencing adapters, amplified by PCR, purified, pooled, and size-selected. Libraries passing quality control were sequenced using an Illumina paired-end sequencing platform. A rice control was included to monitor enzyme digestion efficiency and library construction quality.

### 2.4. Sequencing Data Processing and SNP Discovery

Raw sequencing reads were filtered to obtain high-quality clean reads. Reads containing adapter sequences were removed, and read pairs with more than 10% undetermined bases were discarded. To reduce potential bias caused by restriction-enzyme residues and low-quality terminal bases, high-quality internal read regions were retained for downstream analysis.

Clean reads were assigned to each sample according to dual-index barcodes. Sequencing quality was evaluated using read number, Q30, GC content, and sequencing depth. In total, 1,295,490,603 clean reads were generated, with an average Q30 value of 93.67% and an average GC content of 44.06%. The average sequencing depth was 100.01× for the parental lines and 30.54× for the RIL individuals.

Clean reads were aligned to the peanut reference genome for SNP discovery. SNP calling was performed using both GATK v.4.6.2.0 [17] and SAMtools v. 1.24 [18], and only the concordant SNPs detected by both methods were retained for downstream analysis. A total of 9,685,875 SNPs were identified across the population. The two parental lines, Luojiangjiwo and Fuhuasheng, contained 7,754,513 and 8,078,755 SNPs, respectively, and the RIL individuals contained an average of 5,283,898.88 SNPs per line.

### 2.5. Marker Filtering and Genotyping

Polymorphic SNPs were classified according to the parental genotypes. Because the mapping population was an inbred population derived from two homozygous parents, markers fitting the aaxbb segregation pattern were selected for genetic map construction. A total of 9492 aaxbb-type markers were initially identified.

Markers were filtered to improve genotyping reliability and map quality. Markers with parental sequencing depth lower than 5× were removed. In the progeny, genotypes supported by sequencing depth lower than 2× were treated as missing. Markers were retained only when genotype data were available in at least 60% of the progeny individuals. Markers showing severe segregation distortion were excluded using chi-square testing. After filtering, 2856 high-quality SNP markers were retained for linkage-map construction.

### 2.6. Genetic Linkage Map Construction

A high-density genetic linkage map was constructed using HighMap (http://highmap.biomarker.com.cn, accessed on 9 July 2026) [19]. Pairwise recombination fractions and modified logarithm of odds values were calculated among markers. Markers were assigned to linkage groups according to linkage relationships, and markers with weak linkage to all other markers were removed. Marker order within each linkage group was estimated iteratively, and genotyping errors were corrected during map construction. Recombination fractions were converted into genetic distances using the Kosambi mapping function.

The final linkage map contained 2646 markers distributed across 20 linkage groups, corresponding to the 20 peanut chromosomes. The total map length was 1338.86 centimorgans (cM), with an average marker interval of 0.51 cM. The proportion of gaps smaller than or equal to 5 cM was 98.39%, and the maximum gap was 19.05 cM. The average completeness of mapped marker genotypes was 96.06%.

### 2.7. QTL Analysis

QTL mapping was performed using the R/qtl package [20]. The final genetic linkage map and phenotypic data were integrated for QTL detection. Composite interval mapping was used to identify genomic regions associated with each trait. For each detected QTL, the linkage group, peak position, flanking markers, LOD score, additive effect, and phenotypic variation explained were estimated.

Genome-wide significance thresholds were estimated by permutation testing. QTL exceeding the permutation-derived threshold were considered significant, whereas loci detected at relaxed LOD thresholds were treated as suggestive QTL. QTL names were assigned according to the associated trait and chromosome or linkage group. Physical intervals corresponding to QTL confidence intervals were identified by anchoring flanking markers to the peanut reference genome.

### 2.8. Candidate Gene Identification and Functional Annotation

Genes located within each QTL physical interval were extracted from the peanut reference genome annotation. Candidate genes were functionally annotated using public protein and pathway databases, including Nr, Swiss-Prot, GO, KEGG, COG, KOG, Pfam, and TrEMBL. Functional descriptions were used to identify genes potentially associated with plant architecture, pod development, kernel development, seed size, cell expansion, hormone signaling, carbohydrate metabolism, lipid metabolism, and stress-response pathways.

The number of annotated genes varied among QTL intervals. Candidate-gene information was summarized for each QTL, and genes with plausible functional relationships to the corresponding trait were prioritized for further validation.

### 2.9. Statistical Analysis

Phenotypic data were summarized using descriptive statistics, including mean, range, standard deviation, and coefficient of variation. Trait distributions were examined to assess phenotypic variation among the RILs. Correlation analysis among traits was performed to evaluate relationships between plant architecture, pod, and kernel traits. QTL mapping was conducted using the final genetic map and trait values. The percentage of phenotypic variation explained by each QTL was calculated based on the QTL model.

## 3. Results

### 3.1. Phenotypic Variation in the RIL Population

**Figure 1:**
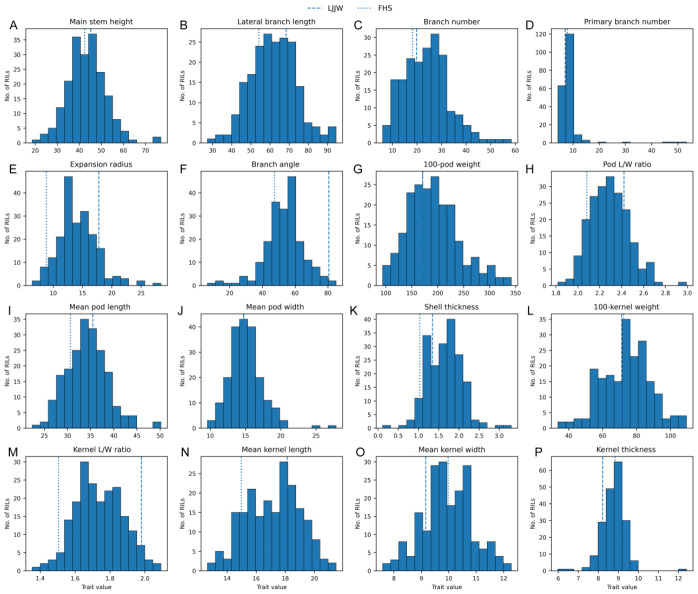
Phenotypic distribution of plant architecture, pod, and kernel traits in the peanut recombinant inbred line population. Histograms show the distribution of trait values among 200 recombinant inbred lines derived from Luojiangjiwo × Fuhuasheng. Dashed and dotted vertical lines indicate the parental values of Luojiangjiwo (LJJW) and Fuhuasheng (FHS), respectively. Panels represent: (**A**) main stem height, (**B**) lateral branch length, (**C**) branch number, (**D**) primary branch number, (**E**) expansion radius, (**F**) branch angle, (**G**) 100-pod weight, (**H**) pod length/width ratio, (**I**) mean pod length, (**J**) mean pod width, (**K**) shell thickness, (**L**) 100-kernel weight, (**M**) kernel length/width ratio, (**N**) mean kernel length, (**O**) mean kernel width, and (**P**) kernel thickness.

Substantial phenotypic variation was observed among the 200 recombinant inbred lines for plant architecture, pod, and kernel traits (Figure 1 and Table S1). The parental lines also differed for several key traits, consistent with their contrasting plant-type and pod-size characteristics described for Luojiangjiwo and Fuhuasheng. Luojiangjiwo showed a larger expansion radius and branch angle than Fuhuasheng, whereas Fuhuasheng had a smaller expansion radius and branch angle, reflecting the contrast between sprawling and erect growth types.

Among plant architecture traits, main stem height ranged from 18.67 to 77.00, with a mean of 43.55 and a coefficient of variation of 20.09%. Lateral branch length ranged from 27.33 to 94.50, with a mean of 61.82 and a coefficient of variation of 19.24%. Branch number showed wider variation, ranging from 6.00 to 58.40, with a coefficient of variation of 38.44%. The primary branch number had the highest relative variation among the recorded traits, with a coefficient of variation of 65.93%, indicating extensive segregation in branch-related traits within the RIL population. Expansion radius and branch angle also showed broad variation, with values ranging from 6.40 to 28.40 and 6.40 to 85.00, respectively.

Pod-related traits also showed marked variation among the RILs. The 100-pod weight ranged from 94.04 to 341.89, with a mean of 192.46 and a coefficient of variation of 25.52%. Mean pod length ranged from 22.38 to 50.18 mm, while mean pod width ranged from 9.60 to 27.84 mm. Shell thickness was recorded for 195 RILs and ranged from 0.11 to 3.30 mm, with a mean of 1.65 mm.

Kernel-related traits displayed moderate to large variation. The 100-kernel weight ranged from 33.64 to 109.42, with a mean of 73.77 and a coefficient of variation of 19.23%. Mean kernel length ranged from 12.64 to 21.50 mm, and mean kernel width ranged from 7.59 to 12.28 mm. Kernel thickness was available for 193 RILs and ranged from 5.98 to 12.40 mm. The wide phenotypic ranges observed across the RIL population indicate that this population is suitable for QTL mapping of plant architecture, pod, and kernel traits.

**Figure 2:**
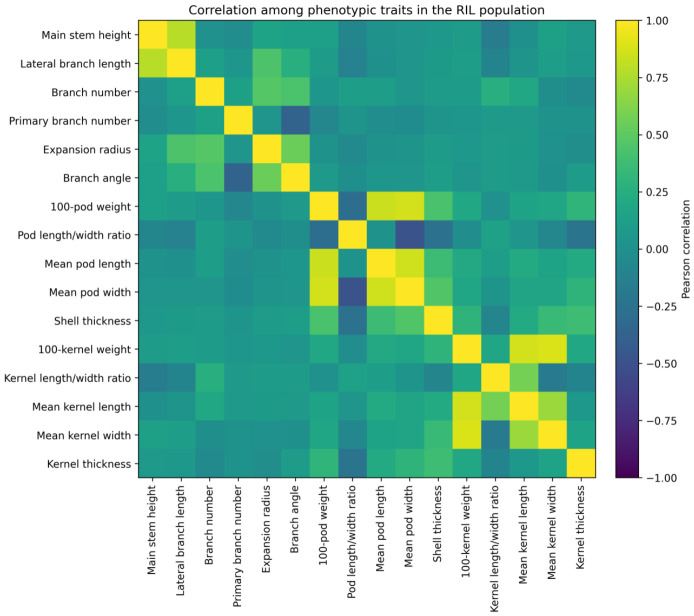
Pearson correlation analysis among plant architecture, pod, and kernel traits in the peanut recombinant inbred line population. The heatmap shows pairwise Pearson correlation coefficients among the 16 measured traits in the 200 recombinant inbred lines.

Pearson correlation analysis revealed clear relationships among plant architecture, pod, and kernel traits (Figure 2 and Table S2). Main stem height was strongly and positively correlated with lateral branch length (r = 0.787), indicating coordinated variation between vertical growth and lateral vegetative growth. Expansion radius was positively correlated with branch angle (r = 0.548), branch number (r = 0.471), and lateral branch length (r = 0.445), suggesting that canopy spread was associated with both branch elongation and branch orientation. Branch number was also positively correlated with branch angle (r = 0.434), whereas primary branch number was negatively correlated with branch angle (r = −0.360).

Strong positive correlations were detected among pod-size traits. The 100-pod weight showed a strong correlation with mean pod width (r = 0.866) and mean pod length (r = 0.840), while mean pod length also demonstrated a strong correlation with mean pod width (r = 0.855). These relationships indicate that heavier pods were generally associated with both greater pod length and greater pod width. Shell thickness was moderately correlated with mean pod width (r = 0.457) and 100-pod weight (r = 0.426). In contrast, pod length/width ratio was negatively correlated with mean pod width (r = −0.485) and 100-pod weight (r = −0.278), indicating that more elongated pods tended to be narrower and relatively lighter.

Kernel-size traits also showed strong internal correlations. The 100-kernel weight was strongly correlated with mean kernel width (r = 0.885) and mean kernel length (r = 0.866), while mean kernel length and mean kernel width were also positively correlated (r = 0.695). Kernel length/width ratio was positively correlated with mean kernel length (r = 0.580) but negatively correlated with mean kernel width (r = −0.179), indicating that kernel shape was influenced more strongly by variation in kernel length than by kernel width. Overall, the wide phenotypic variation and strong correlations among yield-related traits indicate that this RIL population is suitable for QTL mapping of plant architecture, pod, and kernel traits.

### 3.2. Construction and Quality Evaluation of the Genetic Linkage Map

A high-density genetic linkage map was constructed using SNP markers obtained from the RIL population. After marker filtering and linkage analysis, 2646 SNP markers were assigned to 20 linkage groups, corresponding to the 20 chromosomes of cultivated peanut. The total genetic length of the map was 1338.86 cM, and the average marker interval across the whole map was 0.51 cM, indicating high marker density.

**Figure 3:**
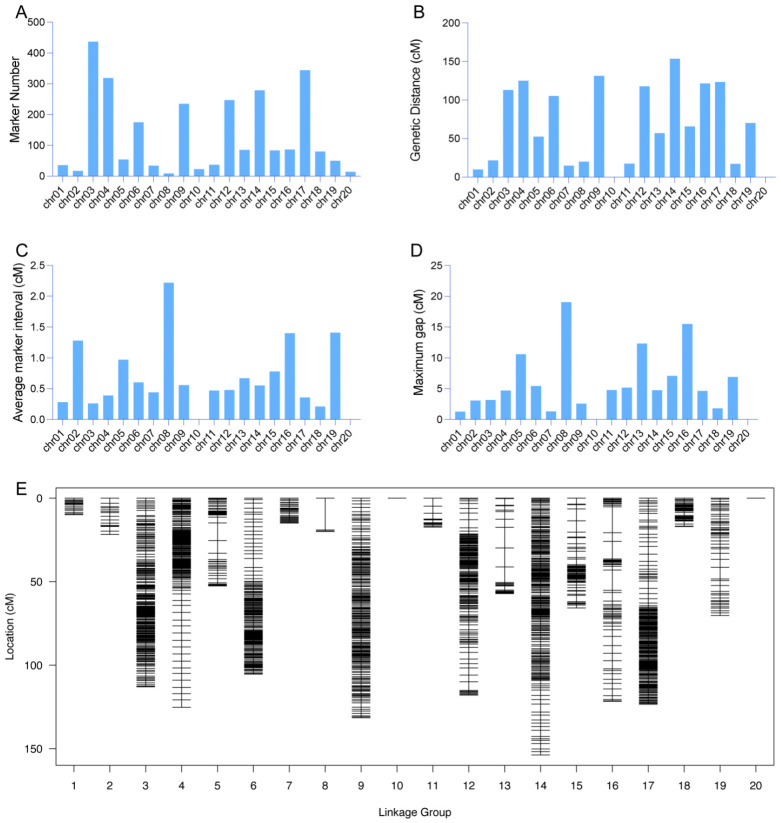
Construction and quality evaluation of the genetic linkage map in the peanut recombinant inbred line population. (**A**) Number of mapped SNP markers on each linkage group. (**B**) The genetic length of each linkage group. (**C**) Average marker interval on each linkage group. (**D**) Maximum marker gap on each linkage group. (**E**) Genetic linkage map showing the order and genetic positions of mapped SNP markers across the 20 linkage groups of cultivated peanut. Marker positions are shown in centimorgans (cM).

Marker number varied among linkage groups (Figure 3A and Table S3). The largest number of markers was mapped to chr03, which contained 437 markers, followed by chr17 with 344 markers, chr04 with 319 markers, chr14 with 279 markers, and chr12 with 247 markers. In contrast, fewer markers were mapped to chr08, chr10, and chr20, which contained 9, 23, and 14 markers, respectively. Genetic length also differed among linkage groups, ranging from 0.00 cM on chr10 and chr20 to 153.82 cM on chr14. Other relatively long linkage groups included chr09 (131.52 cM), chr04 (125.25 cM), chr17 (123.45 cM), chr16 (121.65 cM), and chr12 (117.94 cM).

The linkage map showed generally good marker coverage (Figure 3B). Across the genome, 2609 of 2626 marker intervals were ≤5 cM, representing 99.35% of all intervals. The maximum gap was 19.05 cM, observed on chr08 (Figure 3D). Most linkage groups had maximum gaps below 10 cM, and several linkage groups showed complete or near-complete coverage with all intervals ≤5 cM. The average missing genotype rate across all mapped markers was 3.94%, indicating high marker completeness in the final map. Together, the high marker density, low missing genotype rate, and high proportion of short marker intervals indicate that the genetic map was suitable for downstream QTL analysis.

**Figure 4:**
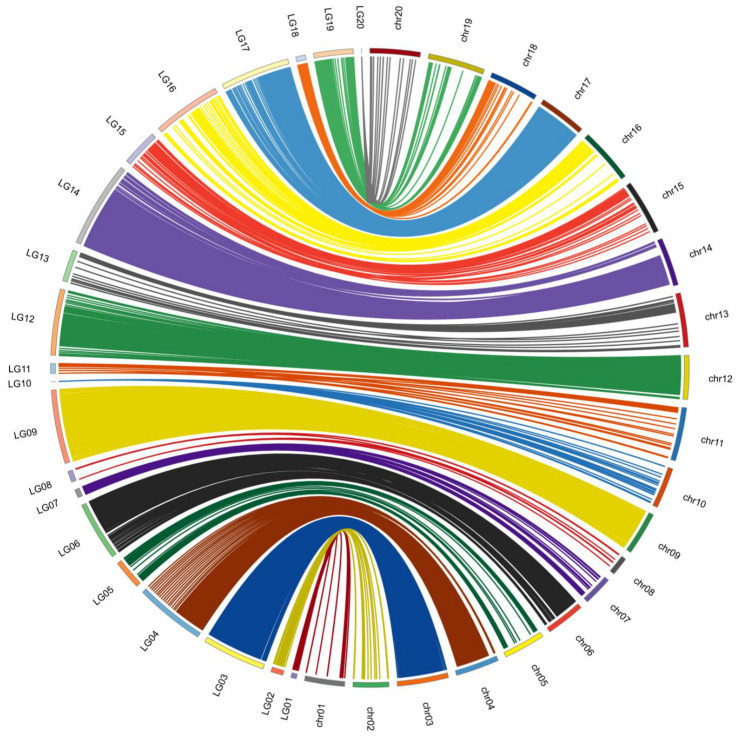
Genetic map and genome collinear diagram. Linkage groups (LG01–LG20) are shown on one side according to their genetic positions (cM), while the corresponding reference chromosomes (Chr01–Chr20) are displayed according to their physical positions (Mb). Each connecting line links the genetic position of an SNP marker on the linkage map to its corresponding physical position on the reference genome.

The complete linkage map showed the order and genetic positions of mapped SNP markers across all 20 linkage groups, while the circos plot showed their genome-wide physical distribution across the peanut chromosomes (Figure 4). Marker positions were generally well distributed across most linkage groups, although several chromosomes showed lower marker density. The integration of linkage-map positions with physical marker distribution confirmed that the final map provided genome-wide marker coverage suitable for QTL analysis.

### 3.3. QTL Mapping for Plant Architecture and Kernel-Related Traits

**Figure 5:**
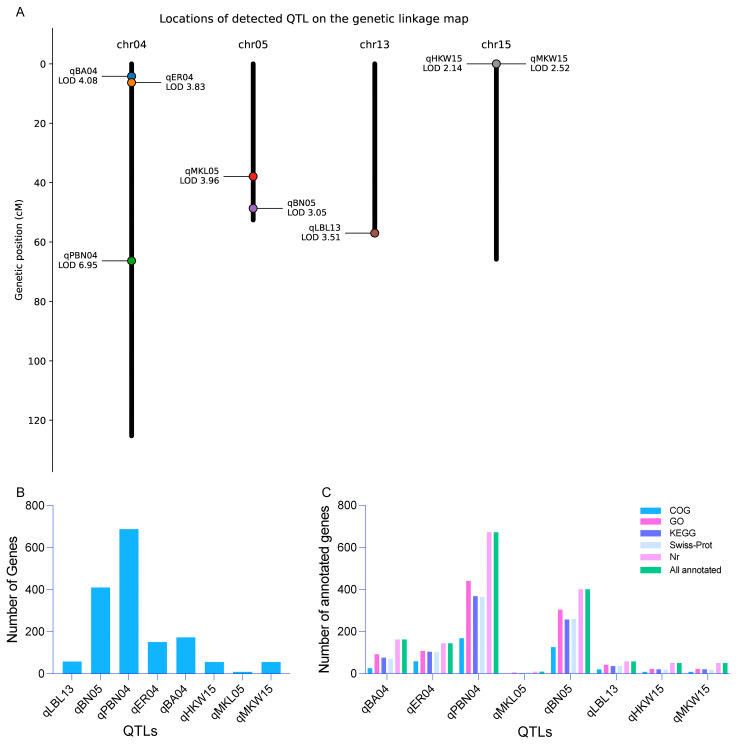
Genomic locations and candidate-gene annotation summary of QTL detected for plant architecture and kernel-related traits in the peanut recombinant inbred line population. (**A**) Locations of the eight detected QTL on the genetic linkage map. QTL were distributed on chr04, chr05, chr13, and chr15. QTL names and maximum LOD scores are shown next to their corresponding genetic positions. Marker positions are shown in centimorgans (cM). (**B**) Number of genes located within each QTL physical interval. (**C**) Functional annotation summary of genes within each QTL interval based on COG, GO, KEGG, Swiss-Prot, Nr, and integrated annotation databases. The qPBN04 interval contained the largest number of genes, whereas qMKL05 contained the fewest genes and represented the narrowest candidate interval.

Composite interval mapping identified eight QTL associated with plant architecture and kernel-related traits in the peanut RIL population. These QTL were distributed on four linkage groups, including chr04, chr05, chr13, and chr15. Five QTL were associated with plant architecture traits, including lateral branch length, branch number, primary branch number, expansion radius, and branch angle, whereas three QTL were associated with kernel-related traits, including 100-kernel weight, mean kernel length, and mean kernel width (Figure 5A; Table S4).

For plant architecture traits, three QTL were detected on chr04. The QTL qBA04 for branch angle was mapped to 4.002–4.367 cM, with a maximum LOD score of 4.077 and phenotypic variation explained as 4.324% (Figure S1). The QTL qER04 for expansion radius was located nearby on chr04 at 6.164–6.444 cM, with a maximum LOD score of 3.828 and phenotypic variation explained of 6.170% (Figure S2). A third QTL on chr04, qPBN04, was associated with primary branch number and mapped to 64.235–68.411 cM. This locus had the highest LOD score among all detected QTL, with a maximum LOD of 6.951, although it explained 3.957% of the phenotypic variation (Figure S3).

**Figure 6:**
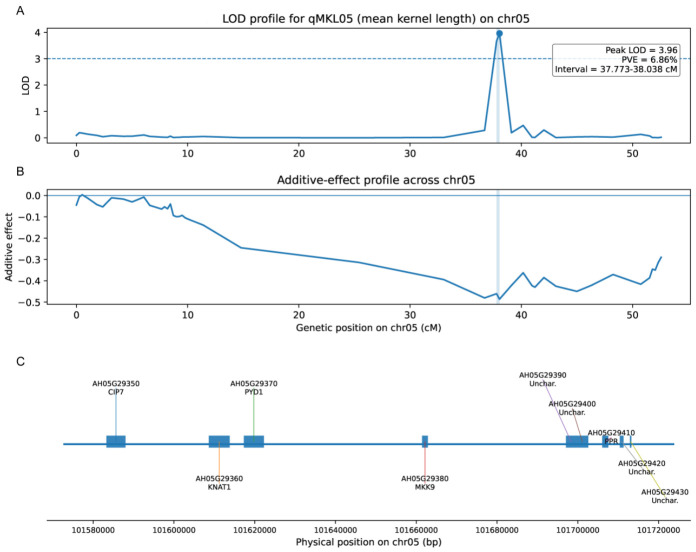
LOD profile, additive effect, and candidate genes for qMKL05 associated with mean kernel length. (**A**) LOD profile of mean kernel length on chr05. The shaded region indicates the qMKL05 confidence interval, and the dashed horizontal line indicates the LOD threshold used for QTL detection. (**B**) Additive-effect profile across chr05. (**C**) Physical distribution of the nine genes located within the qMKL05 interval on chr05: 101,572,621–101,723,856. Gene IDs and abbreviated functional annotations are shown above or below the physical interval.

Two additional plant architecture QTL were detected on chr05 and chr13. For kernel-related traits, one QTL was detected on chr05 and two were detected on chr15. The QTL qMKL05, associated with mean kernel length, was mapped to a narrow interval of 37.773–38.038 cM on chr05, with a maximum LOD score of 3.958 and phenotypic variation explained of 6.861% (Figure 6A,B). Two QTLs on chr15, qHKW15 and qMKW15, were associated with 100-kernel weight and mean kernel width, respectively. Both were located at 0.000 cM and corresponded to the same physical interval on chr15. qHKW15 had a maximum LOD score of 2.140 and explained 5.328% of the phenotypic variation, whereas qMKW15 had a maximum LOD score of 2.518 and explained 4.385% of the phenotypic variation (Figures S5 and S6).

**Figure 7:**
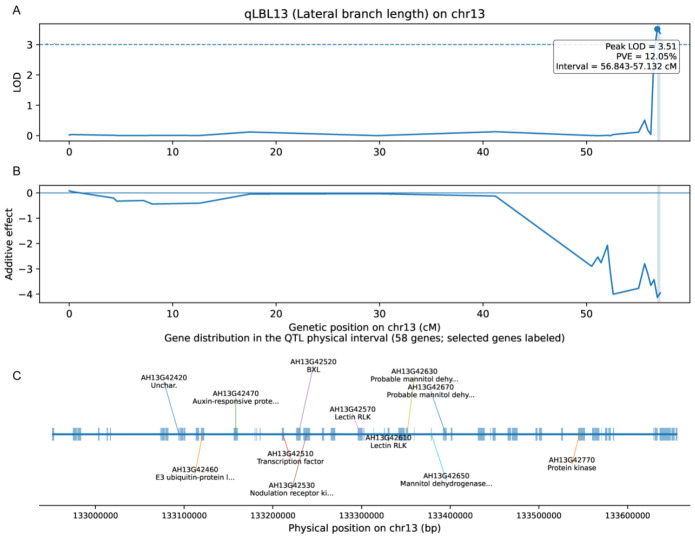
LOD profile, additive effect, and candidate genes for qLBL13 associated with lateral branch length. (**A**) LOD profile of lateral branch length on chr13. The dashed horizontal line indicates the LOD threshold used for QTL detection, and the shaded region indicates the qLBL13 interval. The peak LOD score was 3.51, and the locus explained 12.05% of the phenotypic variation. (**B**) Additive-effect profile across chr13. (**C**) Physical distribution of genes within the qLBL13 interval on chr13. Genes located within the interval are shown as ticks or blocks, and selected annotated genes are labeled. The qLBL13 interval contained 58 genes, including candidates associated with transcriptional regulation, receptor-like kinase signaling, carbohydrate metabolism, and protein kinase activity.

The QTL qBN05, associated with branch number, was mapped to 44.996–52.301 cM on chr05, with a maximum LOD score of 3.046 and phenotypic variation explained as 8.150% (Figure S4). The QTL qLBL13, associated with lateral branch length, was mapped to 56.843–57.132 cM on chr13, with a maximum LOD score of 3.508. This locus explained 12.050% of the phenotypic variation, representing the highest PVE among the detected QTL (Figure 7A,B).

All detected QTL showed negative additive effects, ranging from −0.190 for qMKW15 to −4.129 for qLBL13. Under the genotype coding used in the QTL analysis, the indicates a consistent direction of allelic effects across the detected loci. However, the parental origin of favorable alleles should be interpreted according to the marker coding scheme. Overall QTL locations are summarized in Figure 5A, while chromosome-level LOD profiles, additive-effect profiles, and gene-distribution plots for individual loci are provided in Figure 6, Figure 7 and Figures S1–S6.

### 3.4. Candidate-Gene Identification and Prioritization Within QTL Intervals

Genes located within the physical intervals of the eight detected QTL were extracted and functionally annotated. The physical intervals differed substantially in size and gene content, with the number of genes ranging from 9 in qMKL05 to 688 in qPBN04 (Figure 5B; Table S5). Functional annotation coverage was generally high across QTL intervals, with most genes annotated in at least one database, including COG, GO, KEGG, Swiss-Prot, Nr, and integrated annotation databases (Figure 5C; Tables S5 and S6).

The qMKL05 interval for mean kernel length represented the most clearly resolved candidate region. This interval spanned chr05: 101,572,621–101,723,856 and contained only nine genes, all of which had integrated functional annotations (Figure 6C). Several genes in this interval had annotations consistent with developmental regulation and signaling. *AH05G29360* encodes a knotted-1-like homeobox protein, *AH05G29380* encodes mitogen-activated protein kinase kinase 9, *AH05G29350* encodes COP1-interacting protein 7, and *AH05G29410* encodes a pentatricopeptide repeat-containing protein. Because kernel length is likely influenced by developmental regulation, cell expansion, and signal transduction, these genes represent priority candidates for future validation.

The qLBL13 interval for lateral branch length was also prioritized because it explained the largest proportion of phenotypic variation among the detected QTL. This interval spanned chr13: 132,888,902–133,697,055 and contained 58 genes, all of which had integrated functional annotations (Figure 7C). Selected genes within this region were associated with transcriptional regulation, receptor-like kinase signaling, carbohydrate metabolism, and protein kinase activity. These functional categories are consistent with processes expected to influence lateral branch elongation, including transcriptional control, cell expansion, cell wall remodeling, and growth-related signaling.

The shared chr15 interval underlying qHKW15 and qMKW15 contained 56 genes, of which 51 had integrated annotations (Figures S5C and S6C). The co-localization of QTL for 100-kernel weight and mean kernel width suggests that this region may contribute to kernel-size variation. Candidate genes in this interval included *AH15G16520*, encoding receptor-like protein kinase FERONIA; *AH15G16460*, encoding an agamous-like MADS-box protein; AH15G16630, encoding UDP-glucose 6-dehydrogenase; and *AH15G16770*, encoding jasmonate O-methyltransferase. These genes are plausible candidates because their annotations suggest roles in signaling, developmental regulation, carbohydrate metabolism, cell wall precursor biosynthesis, and hormone-related metabolism.

The chr04 architecture-related intervals contained multiple genes with annotations relevant to plant growth and architecture. The qBA04 interval included selected genes related to transcriptional regulation, carbohydrate metabolism, cellulose biosynthesis, kinase activity, and pentatricopeptide repeat proteins (Figure S1C). The qER04 interval included genes associated with phytochrome signaling, cell wall modification, ubiquitin-mediated protein regulation, carbohydrate metabolism, and wall-associated receptor kinases (Figure S2C). The qPBN04 interval was the broadest candidate region and contained 688 genes, making direct candidate resolution more difficult; nevertheless, selected genes in this interval were annotated as abscisic acid receptor-related proteins, FERONIA, transcription factors, and cytoskeleton-associated proteins (Figure S3C).

KEGG pathway enrichment analysis provided additional functional context for genes located within the detected QTL intervals. In total, 302 KEGG pathway records were identified across the eight QTL regions, of which 19 pathways were significant after multiple-testing correction. Several enriched pathways were related to carbohydrate metabolism, amino acid metabolism, lipid metabolism, secondary metabolism, and regulatory processes (Table S8). For plant architecture-related QTL, qER04 showed enrichment for pathways associated with plant circadian rhythm, flavonoid biosynthesis, and phenylpropanoid biosynthesis, whereas qLBL13 was enriched for beta-alanine metabolism, monoterpenoid biosynthesis, glyoxylate and dicarboxylate metabolism, and propanoate metabolism. For kernel-related QTL, qMKL05 showed enrichment for beta-alanine metabolism, pantothenate and CoA biosynthesis, and pyrimidine metabolism, while the shared qHKW15/qMKW15 interval was enriched for biosynthesis of unsaturated fatty acids (Table S7).

Overall, candidate-gene analysis identified several genes with plausible roles in developmental regulation, signal transduction, cell wall modification, cytoskeletal organization, carbohydrate metabolism, and hormone-related pathways. Among the detected loci, qMKL05 is the strongest candidate interval for further fine mapping because it contains only nine genes, while qLBL13 is important because it explained the largest proportion of phenotypic variation. The shared qHKW15/qMKW15 interval may contain genes affecting kernel-size traits. These candidate genes (Table S8) require validation through expression analysis, marker validation, or functional assays.

## 4. Discussion

The RIL population showed broad phenotypic variation for plant architecture, pod, and kernel traits, confirming its suitability for QTL analysis. Importantly, the extensive phenotypic variation observed in this population enabled the simultaneous dissection of multiple plant architecture and kernel traits, providing an opportunity to identify genomic regions controlling these interconnected yield components within a single mapping population. This integrated genetic analysis extends beyond simple trait characterization by providing a framework for understanding the coordinated genetic control of peanut ideotype development. Similar RIL-based approaches have been used to dissect pod, seed, and architecture-related traits in peanut, including pod length, pod width, hundred-pod weight, seed weight, lateral branch angle, plant height, and branch number [13,14,21,22]. The strong positive correlations among pod-size traits and among kernel-size traits in the present population are consistent with previous reports of co-localized or closely linked QTL for related pod and seed traits. For example, Luo et al. identified co-localized QTL for pod length, pod width, and hundred-pod weight on A05 [22], while Fang et al. reported co-localized QTL for pod and kernel traits on A05 and developed a marker associated with kernel weight [10]. The correlations among expansion radius, branch angle, branch number, and lateral branch length also support the view that peanut plant architecture is determined by multiple interconnected components [14,21,23].

The high-density linkage map developed in this study provided a reliable basis for QTL detection. The map contained 2646 SNP markers across 20 linkage groups, with an average marker interval of 0.51 cM. This marker density is comparable with other high-density peanut maps, such as the SLAF-seq map reported by Li et al. [21], which contained 2808 SNP markers with an average interval of 0.47 cM, and the integrated map reported by Miao et al. [24], which contained 3130 markers with an average interval of 0.64 cM. The present map is also denser than earlier SSR-based maps, such as the map of Huang et al. [25], which contained 470 SSR loci with an average interval of approximately 4.0 cM. These comparisons indicate that the SNP-based map generated here had sufficient marker coverage for QTL analysis. More importantly, the high marker density substantially improved mapping resolution, allowing several QTL to be delimited into relatively small physical intervals. This increased resolution strengthened candidate-gene identification and provides a more practical foundation for downstream fine mapping and marker-assisted breeding than earlier low-density linkage maps.

Five QTL associated with plant architecture traits were detected in this study. Three of them were located on chr04, suggesting that this chromosome may contain regions contributing to branch angle, expansion radius, and primary branch number in this population. This agrees with Meng et al. [23], who reported lateral branch angle-related QTL on multiple chromosomes, including A04. However, because studies differ in mapping populations, marker systems, genome assemblies, and trait definitions, these chr04 loci should be considered as potentially related to previously reported architecture regions rather than confirmed identical QTL. The qLBL13 locus for lateral branch length was especially notable because it explained the largest proportion of phenotypic variation among the detected QTL. Its location on chr13 contrasts with previous reports emphasizing B05 and B09 regions for peanut architecture traits [15,21,26], suggesting that additional loci outside the commonly reported B05/B09 regions may contribute to lateral branch elongation in specific genetic backgrounds. These findings not only support the polygenic nature of peanut plant architecture but also demonstrate that the present population captures additional genetic variation beyond the major architecture loci reported previously [27]. In particular, qLBL13 represents the largest-effect locus detected in this study and therefore constitutes a valuable genomic target for improving branch architecture through marker-assisted breeding and future functional validation.

For kernel-related traits, qMKL05 was the most clearly resolved locus because it was mapped to a narrow region on chr05 containing only nine genes. Chromosome A05/Arahy05 has repeatedly been reported as an important region for pod and seed-size traits. Luo et al. mapped major co-localized QTL for pod length, pod width, and hundred-pod weight to A05 [22], Chu et al. reported a conserved major seed-size QTL on A05 [28], and Fang et al. identified stable co-localized pod/kernel QTL and developed a marker for kernel weight on A05 [10]. Therefore, qMKL05 may lie within or near a broader A05/Arahy05 genomic region involved in seed and kernel development. Although further comparative analyses are required to determine whether qMKL05 corresponds to previously reported A05 loci, the principal advance of the present study lies in refining this region to a narrow 0.151 Mb interval containing only nine annotated genes. This considerably reduces the candidate search space and facilitates subsequent gene validation compared with previously reported broad QTL intervals.

The shared chr15 interval associated with qHKW15 and qMKW15 suggests that 100-kernel weight and mean kernel width may be influenced by pleiotropic effects or tightly linked genes. Co-localization of QTL for related pod and seed traits has been frequently reported in peanut [10,13,22]. However, many major pod- and seed-size loci reported previously occur on A05/Arahy05, B04, B06, or B08 rather than chr15. For instance, Miao et al. detected QTL for hundred-pod and hundred-seed weight on seven chromosomes, including major loci on B04 and B08 [24], while Joshi et al. reported overlapping QTL hotspots for several yield-attributing traits [9]. Therefore, the chr15 interval identified here expands the current catalogue of genomic regions controlling kernel traits in cultivated peanut and represents a promising target for evaluating pleiotropic genetic effects influencing kernel size and weight simultaneously.

Candidate-gene annotation highlighted several plausible biological processes underlying the detected QTL, including developmental regulation, signal transduction, cell wall modification, cytoskeletal organization, carbohydrate metabolism, and hormone-related pathways. The KEGG enrichment results further supported the involvement of metabolic and regulatory pathways in these QTL intervals, although pathway enrichment should be interpreted cautiously because some intervals contained relatively small gene sets. Within qMKL05, genes encoding a knotted-1-like homeobox protein, mitogen-activated protein kinase kinase 9, COP1-interacting protein 7, and a pentatricopeptide repeat-containing protein are plausible candidates for kernel-length variation. The presence of a PPR-containing gene is noteworthy because Zhang et al. also reported PPR genes among candidate genes in A05 pod and seed-size hotspot regions [29]. For qLBL13, selected candidate genes were associated with transcriptional regulation, receptor-like kinase signaling, carbohydrate metabolism, protein kinase activity, and cell wall-related processes, which are relevant to branch elongation and plant architecture. Similar functional categories, including hormone signaling, light response, and cell wall development, have been proposed in previous peanut architecture studies [14,21,26]. Rather than simply identifying genes with relevant annotations, the present study prioritized candidates based on both biological function and mapping resolution. This strategy substantially narrows the number of genes requiring experimental validation and provides a focused set of targets for transcriptomic analysis, allelic characterization, and functional verification using reverse genetics approaches.

## 5. Conclusions

The QTL identified here provide useful genomic regions for peanut plant architecture and kernel-trait improvement. qMKL05 is particularly promising because it is located in a chromosome region repeatedly associated with seed-size traits and contains a small number of genes, making it suitable for fine mapping and marker development. qLBL13 is also important because it explained the largest phenotypic contribution among the detected loci and may represent an additional architecture-related region outside the commonly reported B05/B09 loci. However, several limitations should be considered. Some loci were detected under relaxed LOD thresholds and should be regarded as suggestive until validated. In addition, the present analysis appears to be based on a single phenotypic dataset, whereas many robust peanut QTL studies used multi-environment phenotyping to evaluate QTL stability [14,22,24]. Finally, broad intervals such as qPBN04 and qBN05 still contain many genes and require further fine mapping. The candidate genes were ranked based on QTL location and functional annotation instead of sequence variant annotation. Consequently, the identification and functional characterization of causative variations within these genes will necessitate future whole-genome resequencing and variant effect investigations. Subsequent research should integrate multi-environment validation, marker creation, expression profiling, and functional tests to verify the stability and biological activities of these loci. The high-resolution linkage map, revised QTL intervals, and prioritized candidate genes produced in this study collectively establish a significant genomic basis for enhancing the genetic development of plant architecture and kernel characteristics in cultivated peanut.

## Data Availability

Raw sequencing data are available at NCBI under the project number PRJNA1490913. All other data supporting this manuscript are within the text or its Supplementary Files.

## Supplementary Materials

The following supporting information can be downloaded at: https://www.mdpi.com/article/10.3390/genes17070792/s1. Figure S1: LOD profile, additive-effect profile, and gene distribution for qBA04 associated with branch angle on chr04. (A) Chromosome-level LOD profile. The dashed horizontal line indicates the LOD threshold used for QTL detection, and the shaded region indicates the qBA04 interval. (B) Additive-effect profile across chr04. (C) Gene distribution within the corresponding physical interval. All genes are shown as ticks or blocks, and selected annotated genes are labeled; Figure S2: LOD profile, additive-effect profile, and gene distribution for qER04 associated with expansion radius on chr04. (A) Chromosome-level LOD profile. The dashed horizontal line indicates the LOD threshold used for QTL detection, and the shaded region indicates the qER04 interval. (B) Additive-effect profile across chr04. (C) Gene distribution within the corresponding physical interval. All genes are shown as ticks or blocks, and selected annotated genes are labeled; Figure S3: LOD profile, additive-effect profile, and gene distribution for qPBN04 associated with primary branch number on chr04. (A) Chromosome-level LOD profile. The dashed horizontal line indicates the LOD threshold used for QTL detection, and the shaded region indicates the qPBN04 interval. (B) Additive-effect profile across chr04. (C) Gene distribution within the corresponding physical interval. All genes are shown as ticks or blocks, and selected annotated genes are labeled; Figure S4: LOD profile, additive-effect profile, and gene distribution for qBN05 associated with branch number on chr05. (A) Chromosome-level LOD profile. The dashed horizontal line indicates the LOD threshold used for QTL detection, and the shaded region indicates the qBN05 interval. (B) Additive-effect profile across chr05. (C) Gene distribution within the corresponding physical interval. All genes are shown as ticks or blocks, and selected annotated genes are labeled; Figure S5: LOD profile, additive-effect profile, and gene distribution for qHKW15 associated with 100-kernel weight on chr15. (A) Chromosome-level LOD profile. The dashed horizontal line indicates the LOD threshold used for QTL detection, and the shaded region indicates the qHKW15 interval. (B) Additive-effect profile across chr15. (C) Gene distribution within the corresponding physical interval. All genes are shown as ticks or blocks, and selected annotated genes are labeled; Figure S6: LOD profile, additive-effect profile, and gene distribution for qMKW15 associated with mean kernel width on chr15. (A) Chromosome-level LOD profile. The dashed horizontal line indicates the LOD threshold used for QTL detection, and the shaded region indicates the qMKW15 interval. (B) Additive-effect profile across chr15. (C) Gene distribution within the corresponding physical interval. All genes are shown as ticks or blocks, and selected annotated genes are labeled; Table S1: Phenotypic variation and descriptive statistics for plant architecture, pod, and kernel traits in the peanut RIL population; Table S2: Pearson correlation coefficients among plant architecture, pod, and kernel traits in the peanut RIL population; Table S3: Summary statistics of the high-density genetic linkage map constructed for the peanut RIL population; Table S4: Summary of QTL detected for plant architecture and kernel-related traits in the peanut RIL population; Table S5: Physical intervals, gene numbers, and functional annotation statistics for genes within detected QTL regions; Table S6: Genes identified within QTL intervals associated with plant architecture and kernel-related traits; Table S7: KEGG pathway enrichment analysis of genes located within detected QTL intervals; Table S8: Functional annotation details of candidate genes prioritized from detected QTL intervals.
