# Supplementary figures and images for: High-Density Genetic Mapping Identifies QTL and Candidate Genes for Plant Architecture and Kernel Traits in Cultivated Peanut
Source: Genes (Basel). 2026 Jul 12;17(7):792. doi: 10.3390/genes17070792 (PMC13409475; doi:10.3390/genes17070792)

# qBA04 (Branch angle) on chr04

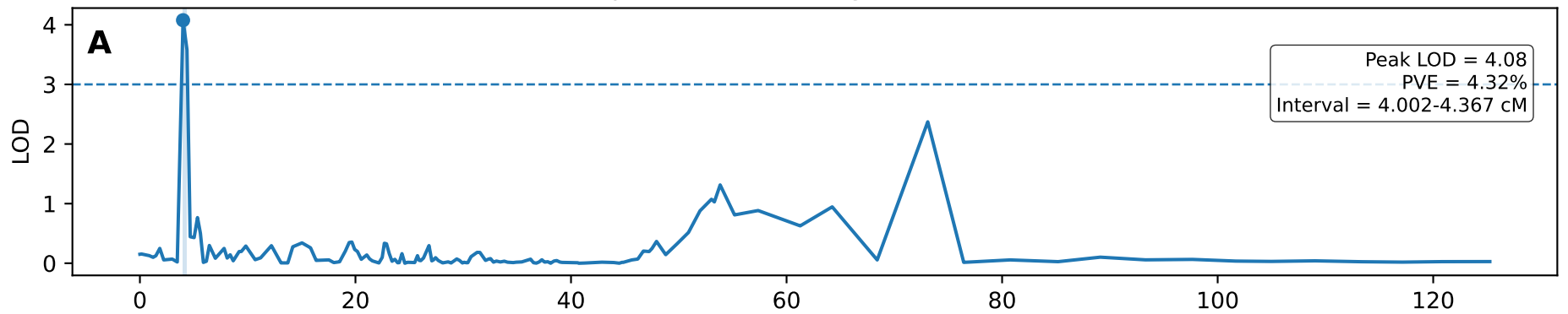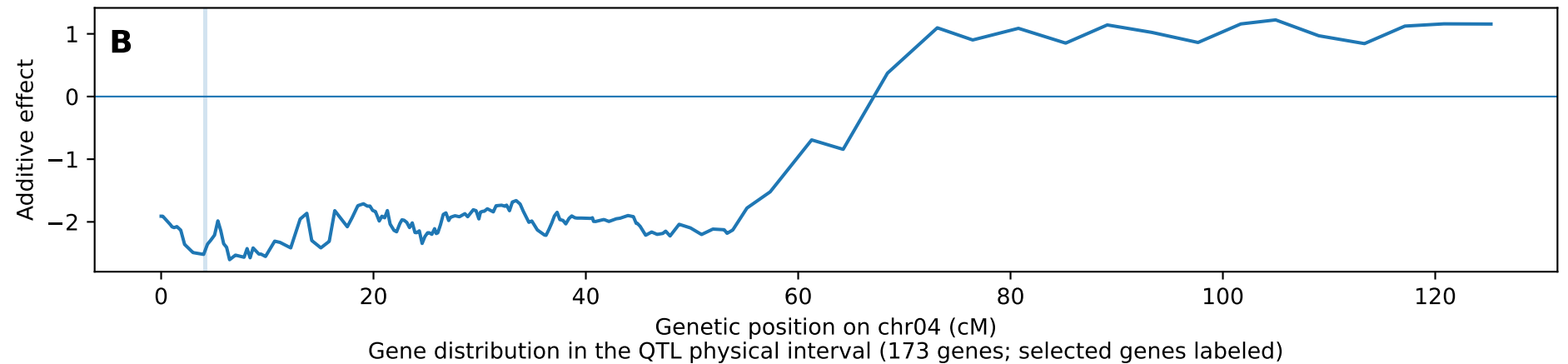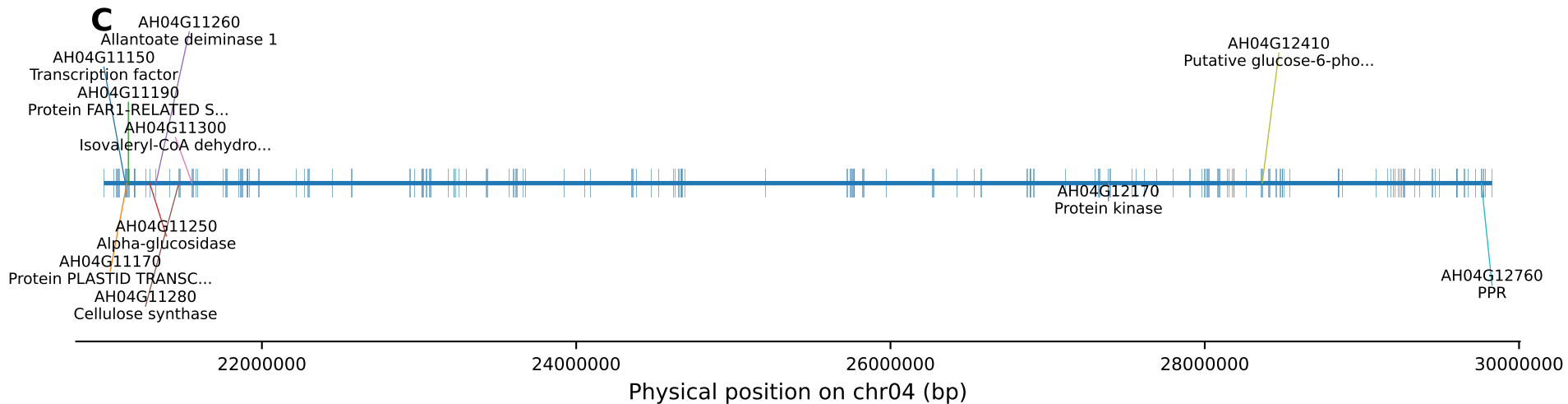

Supplement: Supplementary file 1 [file genes-17-00792-s001.zip › FIgure S1.pdf]

# qER04 (Expansion radius) on chr04

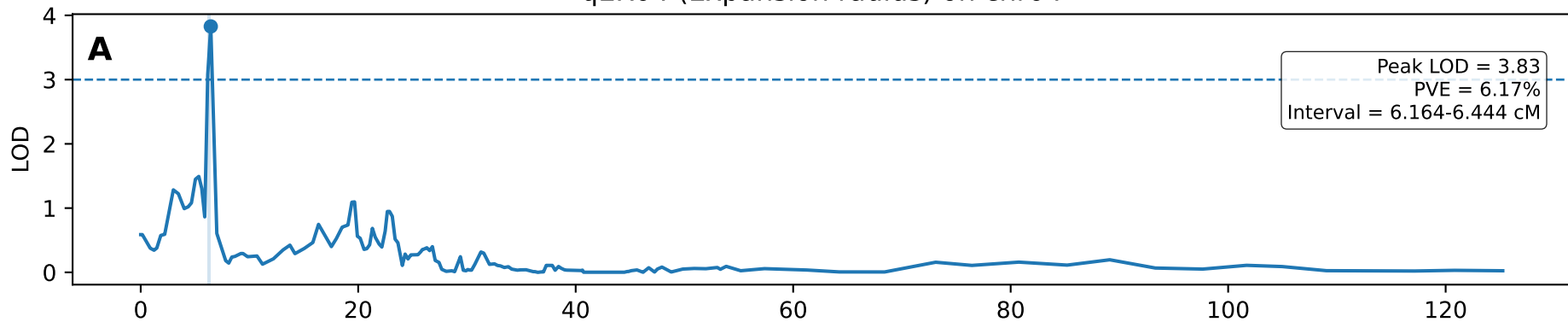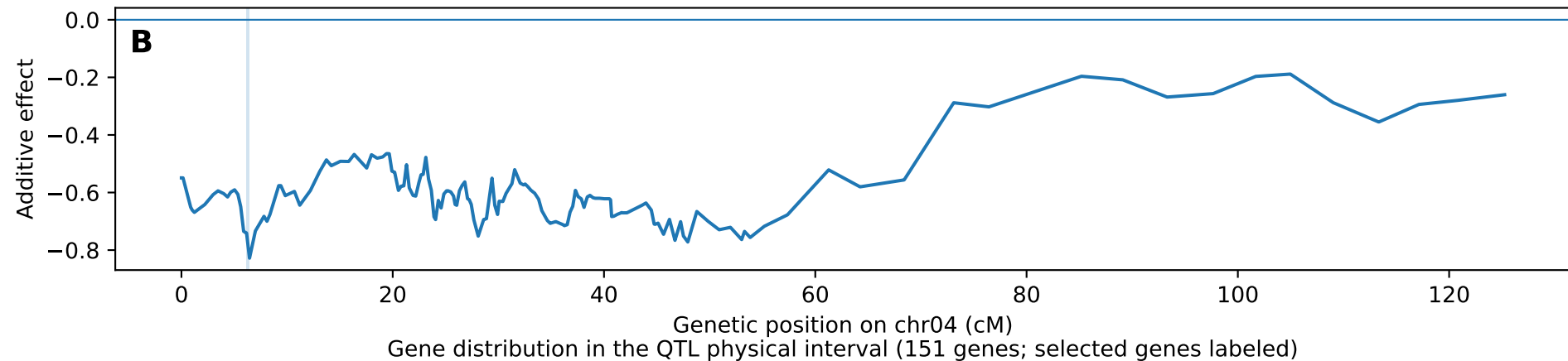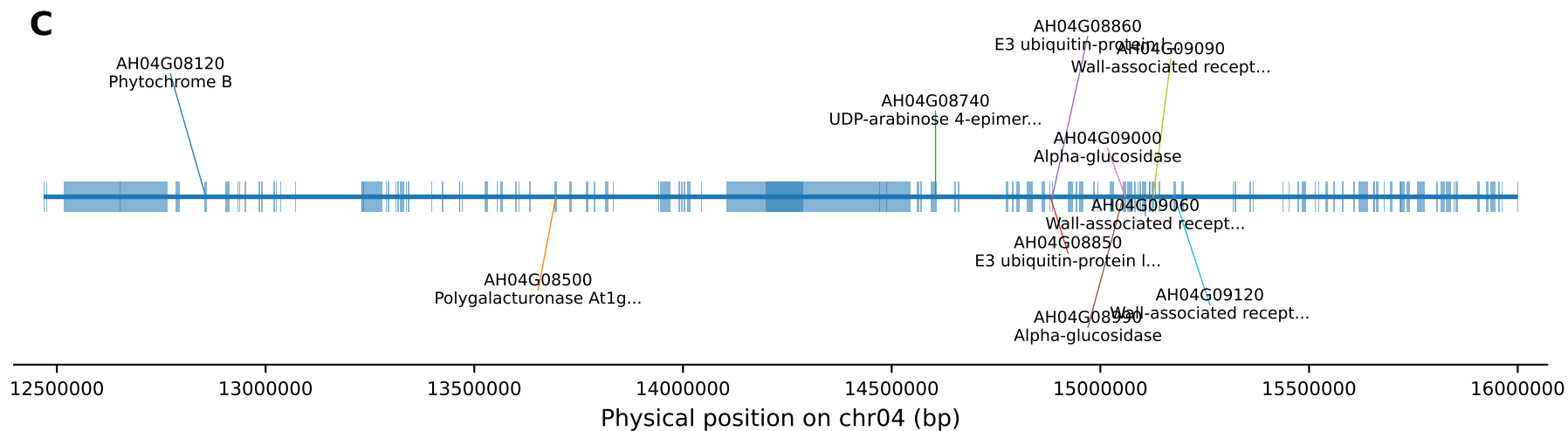

Supplement: Supplementary file 1 [file genes-17-00792-s001.zip › FIgure S2.pdf]

# qPBN04 (Primary branch number) on chr04

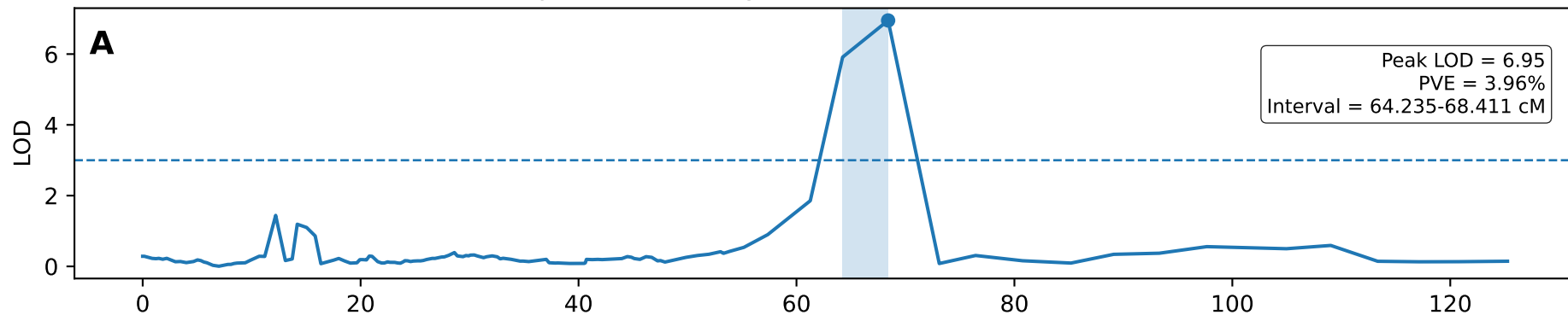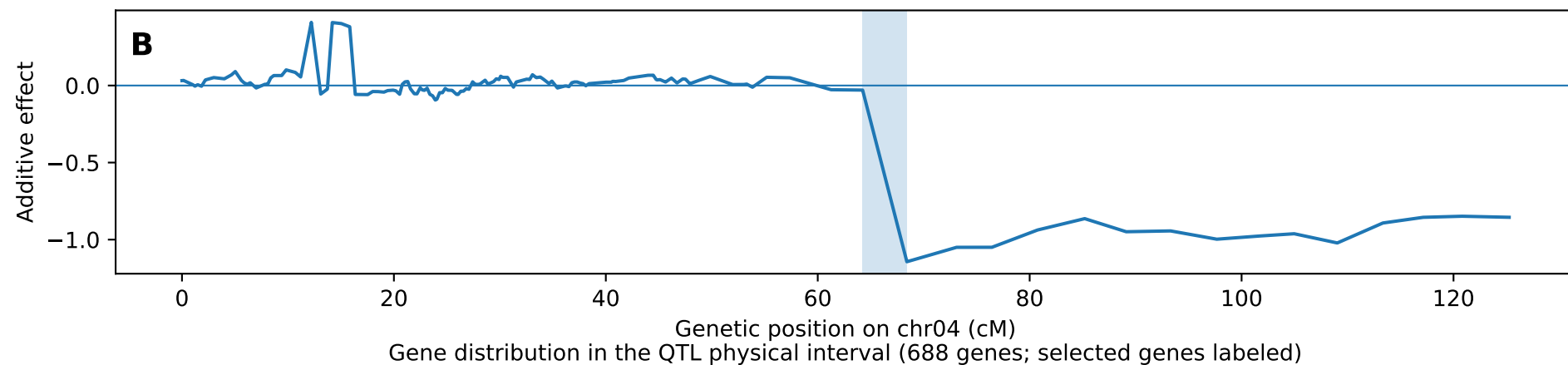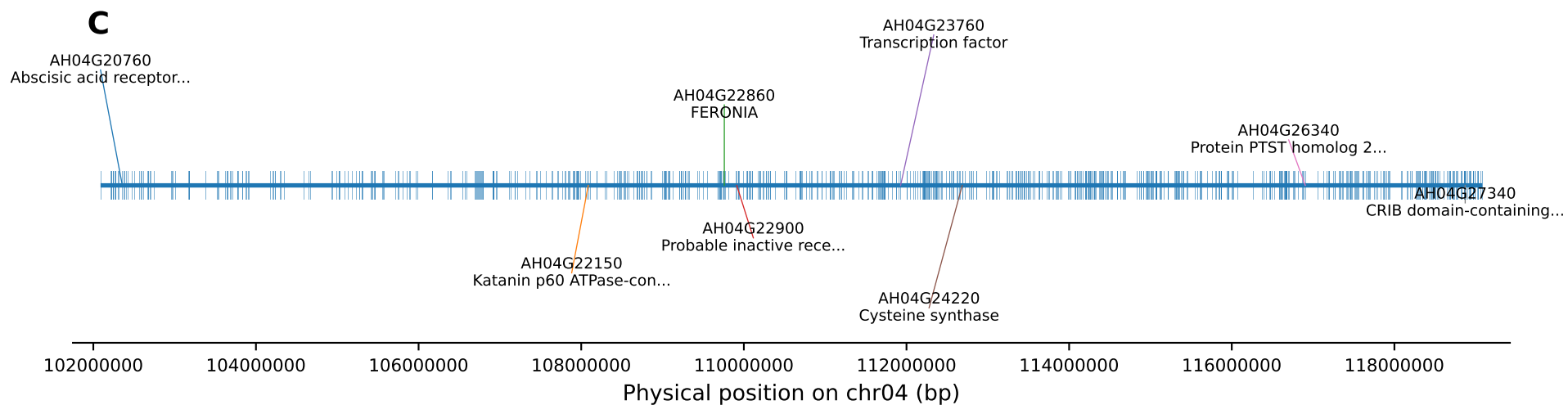

Supplement: Supplementary file 1 [file genes-17-00792-s001.zip › FIgure S3.pdf]

# qBN05 (Branch number) on chr05

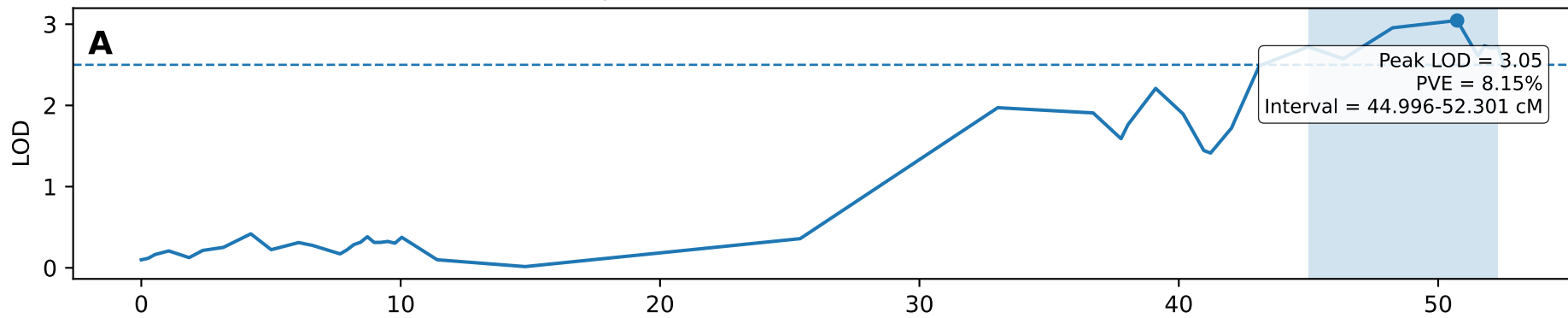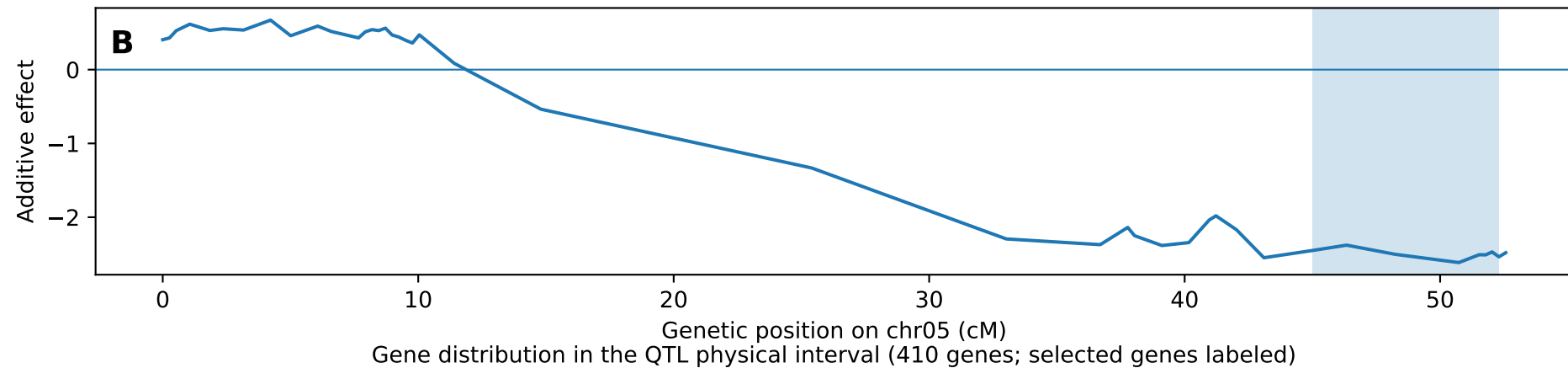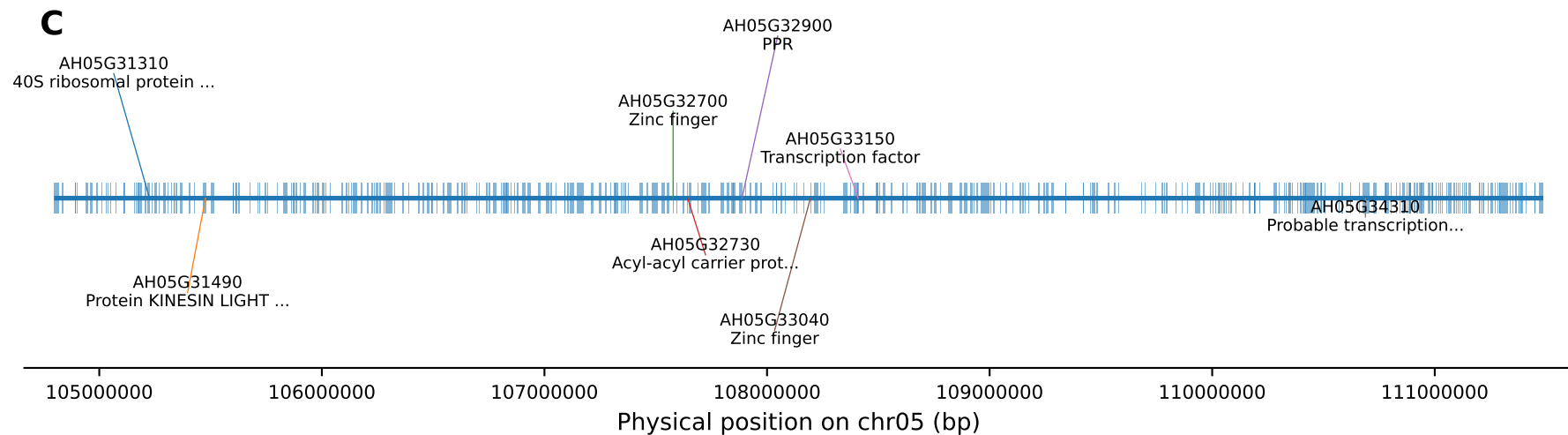

Supplement: Supplementary file 1 [file genes-17-00792-s001.zip › FIgure S4.pdf]

# qHKW15 (100-kernel weight) on chr15

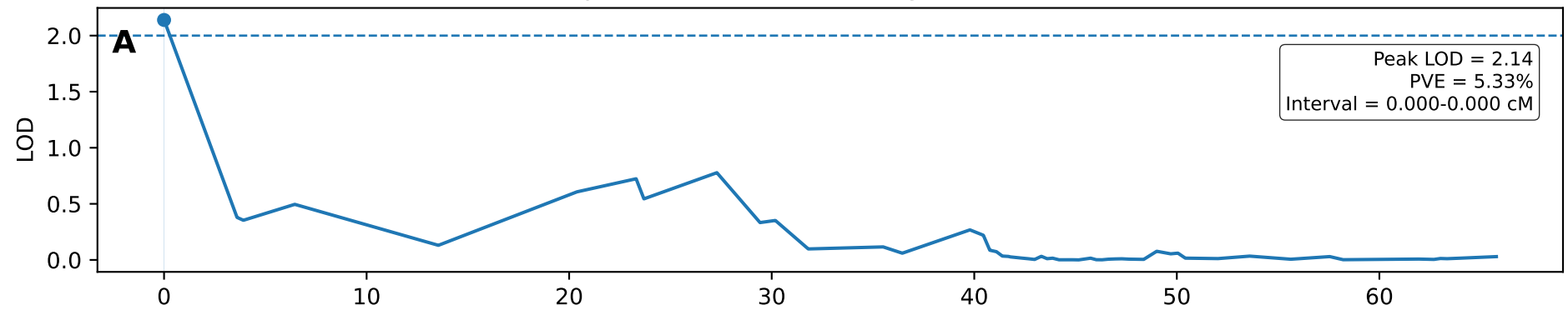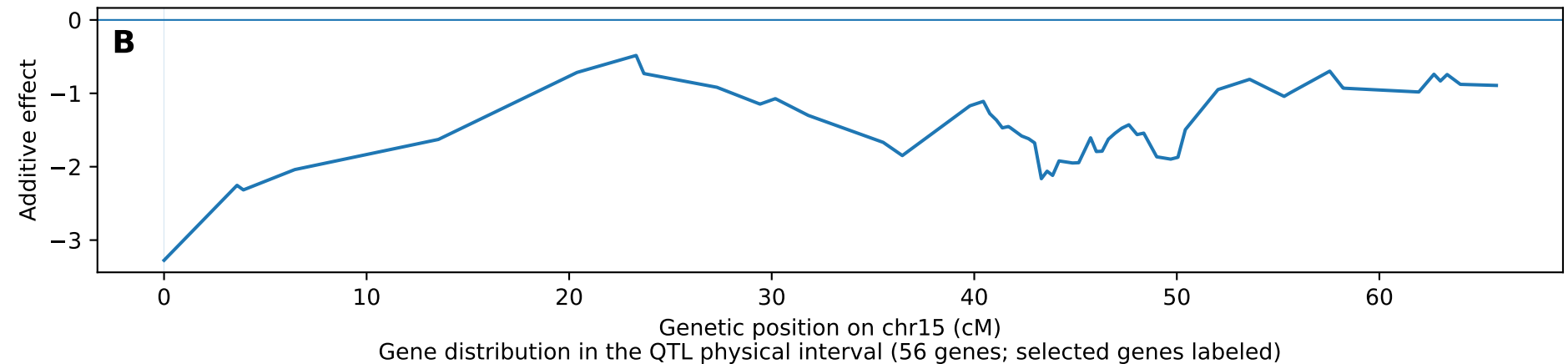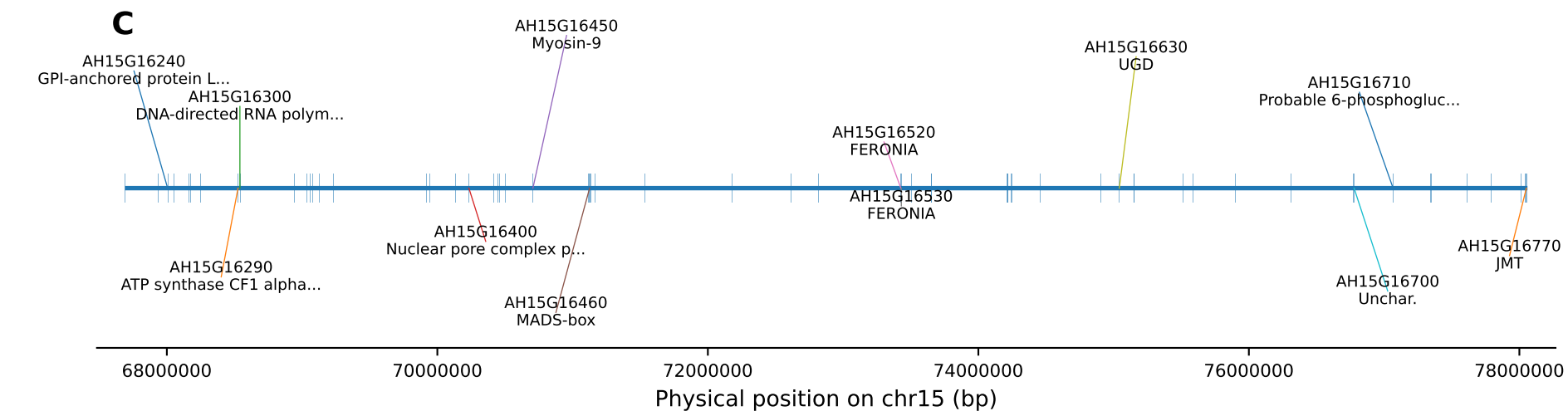

Supplement: Supplementary file 1 [file genes-17-00792-s001.zip › FIgure S5.pdf]

# qMKW15 (Mean kernel width) on chr15

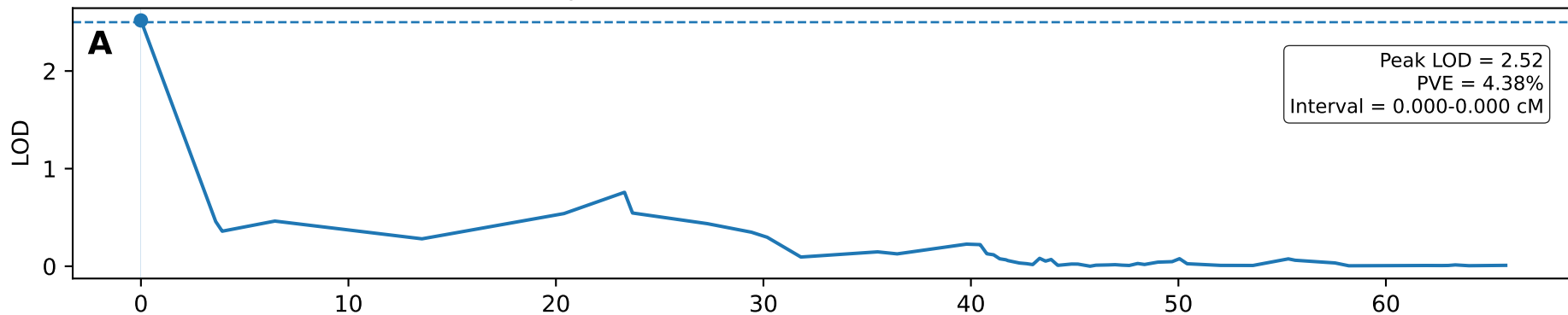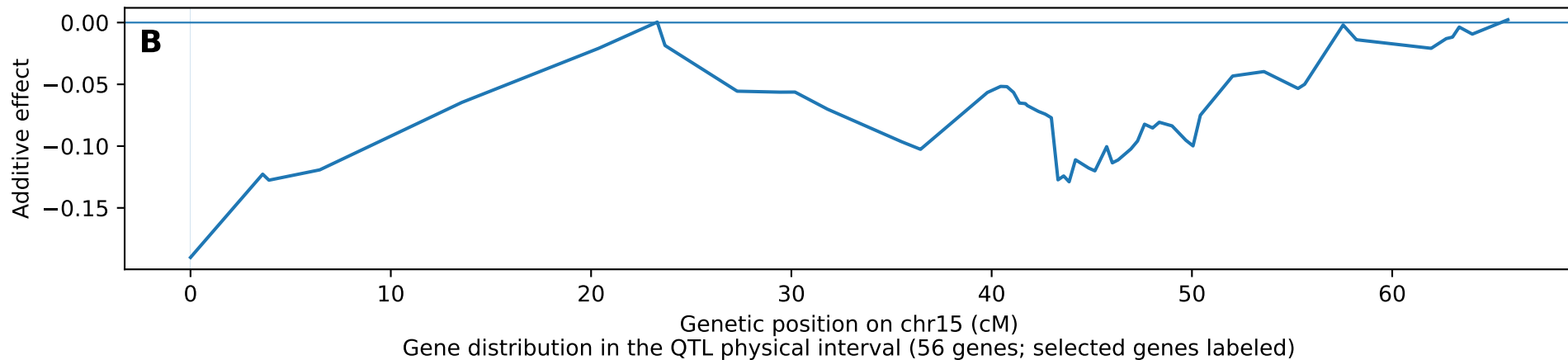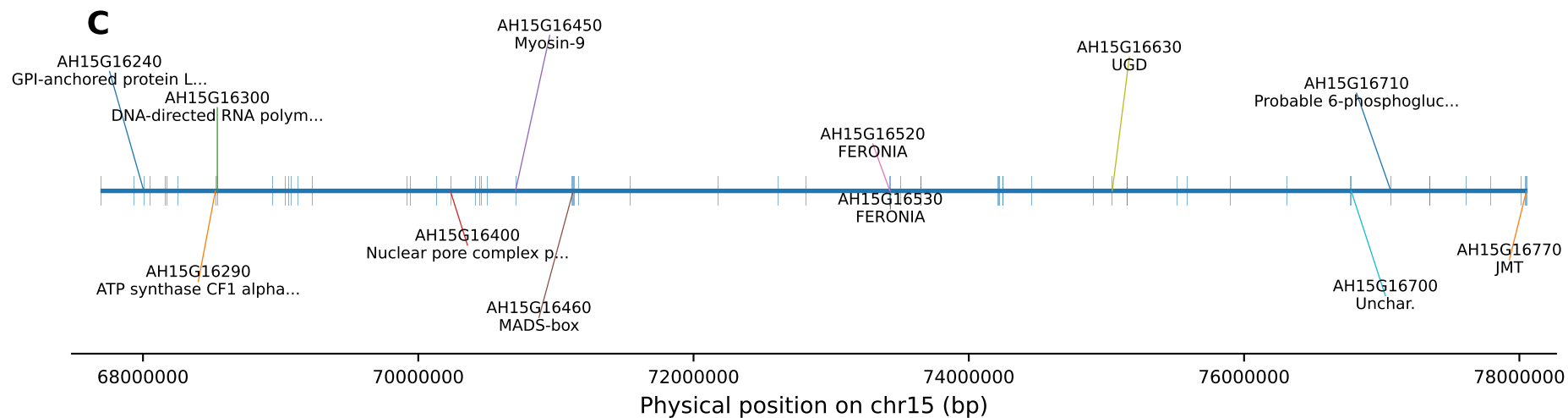

Supplement: Supplementary file 1 [file genes-17-00792-s001.zip › FIgure S6.pdf]
